# Comparative Analysis of Polyphenolic Profile and Chemopreventive Potential of Hemp Sprouts, Leaves, and Flowers of the Sofia Variety

**DOI:** 10.3390/plants13152023

**Published:** 2024-07-23

**Authors:** Agnieszka Galanty, Paulina Juncewicz, Irma Podolak, Karolina Grabowska, Piotr Służały, Paweł Paśko

**Affiliations:** 1 Department of Pharmacognosy, Jagiellonian University Medical College, Medyczna 9, 30-688 Kraków, Poland; paulina.juncewicz@student.uj.edu.pl (P.J.); irma.podolak@uj.edu.pl (I.P.); karolina1.grabowska@uj.edu.pl (K.G.); piotr_s-95@o2.pl (P.S.); 2 Department of Food Chemistry and Nutrition, Jagiellonian University Medical College, Medyczna 9, 30-688 Kraków, Poland; p.pasko@uj.edu.pl

**Keywords:** sprouts, flavonoids, cytotoxic, *Cannabis*, chemoprevention

## Abstract

This study investigates the phytochemical composition and biological activities of hemp (*Cannabis sativa* L.) leaves, flowers’ methanolic extracts from the Sofia variety, and its sprouts cultivated under different light conditions (natural light, darkness, blue, and white LED light for 5, 7, and 9 days). Phytochemical analysis using HPLC identified four key polyphenolic compounds in sprouts’ extracts: chlorogenic, caffeic, and gallic acids, and myricetin, with a predomination of the chlorogenic acid. In contrast, leaves and flowers’ extracts contained cannflavins A and B and chlorogenic, *p*-coumaric, and ferulic acids, with a significant presence of isochlorogenic acid. Antioxidant capacity, assessed by FRAP method, revealed higher antioxidant potential in leaves compared to flowers and sprouts, with sprouts grown under blue and white LED lights exhibiting the highest activity. Cytotoxic activity was evaluated on human colon cancer cell lines (HT29, HCT116, DLD-1) and normal colon epithelial cells (CCD 841 CoN). Results demonstrated significant and selective cytotoxicity against cancer cell lines, with leaves showing more pronounced effects than flowers, and sprouts only moderate activity. All samples revealed an anti-inflammatory effect in vitro. To conclude, sprouts, leaves, and flowers of the Sofia hemp may be considered promising products for chemoprevention in the future.

## 1. Introduction

*Cannabis sativa* L. is a species of Asian origin that has been cultivated since ancient times for commercial, nutritional, and medicinal purposes. It is a dioecious plant belonging to the *Cannabaceae* family. Dependent on the purpose, the plant products are derived from one of the two subspecies, namely *C. sativa* subsp. *sativa* (hemp) or *C. sativa* subsp. *indica* (cannabis). The monoecious specimens are mainly used for extracting oil from the seeds and are an important raw material for fiber production. Inflorescences of female plants, known as marijuana, have in recent years regained medicinal value, especially for the treatment of certain types of epilepsy [1].

Phytochemistry of *Cannabis sativa* L. is centered around unique terpenophenolic compounds called phytocannabinoids, of which delta-9-tetrahydrocannabinol (THC) and cannabidiol (CBD) are the most widely studied. Other important phytochemicals include terpenes present in the essential oil, flavonoids and phenolic acids, protein, polyunsaturated fatty acids in seed oil, vitamins, and inorganic nutrients [2,3]. For this reason, various hemp products such as seeds, leaves, and sprouts are gaining interest as promising functional food candidates [4]. Apart from nutritional value, the health-enhancing qualities of their polyphenolic constituents are becoming increasingly appreciated. Among these, rare flavonoids such as cannflavins, with potent anti-inflammatory activity, hold a special place [4].

Sprouts are interesting examples of food products whose chemopreventive potential has been extensively studied. The phytochemicals present in sprouts tend to be more bioavailable for the human organism, compared to the corresponding mature plants, and represent different chemical classes of compounds with antioxidant, cytotoxic, and anti-inflammatory properties [5]. Among various methods used to increase the contents of phytochemicals in sprouts, their cultivation under different lighting conditions is one of the most interesting. These include the use of natural light, darkness, or LED lights at different wavelengths. A number of studies have reported that modification of lighting conditions results in changes in the content of flavonoids, phenolic acids, and sulfur compounds in sprouts [6,7,8].

There are currently numerous varieties and cultivars of *C. sativa*, obtained through selection to provide a specific content of the psychoactive phytocannabinoid tetrahydrocannabinol (THC), which is legally controlled, and should not exceed 0.2%. Sofia is one of the varieties recently registered and approved for cultivation in Poland, obtained from *C. sativa* subsp. *sativa*. While dried leaves and flowers of this variety are available on the market (for teas), little is known of its phenolic composition and health-promoting potential. Moreover, the data on hemp sprouts are scarce, as only a few studies so far have reported on the process of hemp seed germination and the chemical profile of sprouts, while no information has been provided on their bioactivity.

Therefore, the aim of our study was to investigate the commercially available leaves and flowers of the Sofia variety, as well as sprouts grown under different lighting conditions, with regard to the content of polyphenolic compounds. Moreover, antioxidant, anti-inflammatory, and cytotoxic properties targeting colon cancer cell lines of various origins were investigated to verify the chemopreventive potential of Sofia hemp products.

## 2. Results and Discussion

### 2.1. Quantitative HPLC Analysis

**Figure 1:**
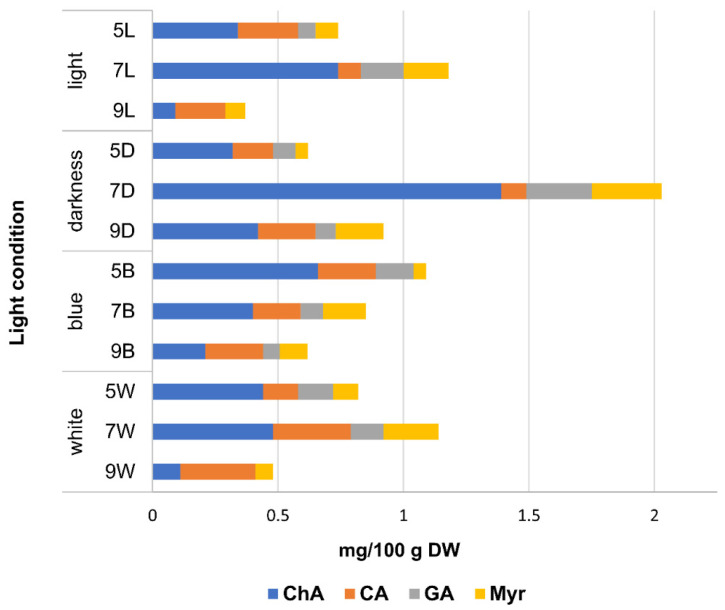
Cumulation dynamics of phenolic acid (ChA, chlorogenic acid; CA, caffeic acid; GA, gallic acid) and flavonoid (Myr, myricetin) content in the sprouts of Sofia hemp variety, grown under different lighting conditions, and harvested at different (5, 7, 9) days after sowing.

In the first stage of the study, quantitative HPLC analysis was carried out on the extracts from Sofia hemp leaves and flowers, as well as seeds and sprouts, grown under different lighting conditions (daylight, darkness, and white and blue LED light). Four polyphenolic compounds were identified in the extracts from the tested sprout samples: chlorogenic acid, caffeic acid, gallic acid, and myricetin (Table 1, Figure S1). Interestingly, only chlorogenic acid was identified in hemp seeds, and this compound predominated in sprouts, accompanied by three other phenolic acids. It was observed that the synthesis of most of the compounds identified in the sprouts increased up to the seventh day of cultivation, after which their content decreased, with the opposite tendency only noted for caffeic acid (Figure 1). Our study is the first to report on the content of phenolic acids in hemp sprouts. Regarding the presence of flavonoids, we noted myricetin for the first time, while no cannflavins were detected in Sofia-variety hemp sprouts’ extracts. This stands in contrast to the results of Werz et al., who reported the presence of both cannflavin A and B in the sprouts of only one of the three Italian hemp varieties tested [4]. Interestingly, the presence of cannflavins was detected in hemp microgreens of the Finola variety [9], in the sprouts of which these compounds were not found [4]. This may suggest that the synthesis of cannflavins requires a longer cultivation time, as microgreens are usually grown for several days, in contrast to sprouts.

A comparison of the content of polyphenolic compounds in sprouts grown under varying light conditions showed interesting differences. Chlorogenic acid synthesis was intensified in total darkness compared to natural light conditions, while its content in sprouts grown under LED light was significantly lower (*p* < 0.05). On the other hand, both LED lights enhanced the synthesis of caffeic acid compared to natural light and to darkness. In a similar study, blue LED light also increased total polyphenolic and total flavonoid contents compared to natural light conditions [6]. Our results indicate that the optimal conditions for sprouts rich in chlorogenic acid, gallic acid, and myricetin appear to be cultivation in the darkness and harvesting on the seventh day after sowing, while for caffeic acid the optimal conditions are cultivation in white light and harvesting on the seventh day.

Four phenolic acids, namely chlorogenic acid, isochlorogenic acid, *p*-coumaric acid, and ferulic acid were identified in the extracts from leaves and flowers of Sofia hemp, with isochlorogenic acid predominating (Table 2, Figures S2 and S3). The flowers of the Sofia hemp variety also contained a high amount of ferulic acid, in contrast to the leaves. Chlorogenic acid was present at similar levels in both leaves and flowers, while *p*-coumaric acid was present in the lowest amounts in both products tested. The predominance of ferulic acid in Sofia flowers is in line with the results of other authors, where the content of this compound ranged from 3 to 35.6 mg/kg DW [10], while no information was found on the presence of isochlorogenic acid.

Isoquercetin and cannflavin A were the two dominant flavonoids present in the leaves and flowers of the Sofia variety. Interestingly, isoquercetin was predominant in the flowers, while cannflavin A in the leaves. The amount of myricetin was six times higher in the flowers than in the leaves, while the content of cannflavin B was comparable in both products, but significantly lower than cannflavin A. Quercetin was only found in small amounts in hemp leaves, but not in flowers. In a similar study, the content of cannflavin A in six hemp varieties ranged from 59.9 ± 3.4 to 318.9 ± 5.8 µg/g, while cannflavin B from 21.0 ± 2.6 to 412.2 ± 4.0 µg/g. Interestingly, the predominance of cannflavin A over B was noted in five out of six examined varieties [11], which is consistent with our results. The amount of isoquercetin in the Sofia flowers is significantly higher compared to the results of other authors, with reported contents ranging from 2.2 to 285.9 mg/kg DW in four hemp varieties from Italy [10]. Myricetin has been reported in *Cannabis sativa* samples consisting of leaves and flowers mixed together [12].

### 2.2. Antioxidant Capacity

Protecting the body from the excess of free radicals is an important element of chemoprevention. Therefore, based on the promising results regarding the flavonoid and phenolic acid contents of the tested hemp products, we decided to determine their antioxidant potential. The germination process significantly increased the antioxidant capacity, as could be seen by comparing the observed levels for sprouts, which ranged from 9.48 to 19.74 μM Fe^2+^/g DW (Table 1), and seeds (1.42 μM Fe^2+^/g DW). Sprouts grown under white and blue LED light showed the highest antioxidant properties, compared to natural light and darkness. Furthermore, the properties of sprouts grown under both LED lights increased with harvest time, while no such effect was observed for sprouts grown under natural light and in darkness. The antioxidant activity of the tested sprouts correlated moderately with chlorogenic acid content (r = 0.63, *p* < 0.05). The results obtained for the sprouts are comparable with those obtained by other authors, who reported high antioxidant potential of the sprouts, measured by DPPH and ORAC, which correlated with total polyphenolic content [3]. Similarly, higher antioxidant properties were reported for the sprouts grown under blue LED light, compared to natural light conditions [6].

Interestingly, the antioxidant capacity of hemp leaves was more than twice that of the flowers (Table 2). Similar results were obtained for three varieties grown in Poland, Białobrzeskie, Tygra, and Henola, the antioxidant potential of whose leaves was higher than that of the flowers. However, this effect depended on the extraction process, as for the same samples extracted with different supercritical CO_2_ process parameters, an inverse relationship was noted [13].

### 2.3. Cytotoxic Activity

**Figure 2:**
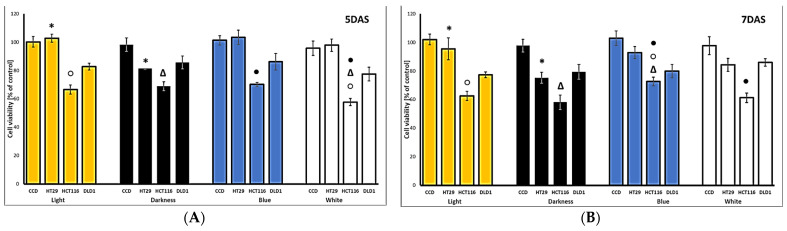

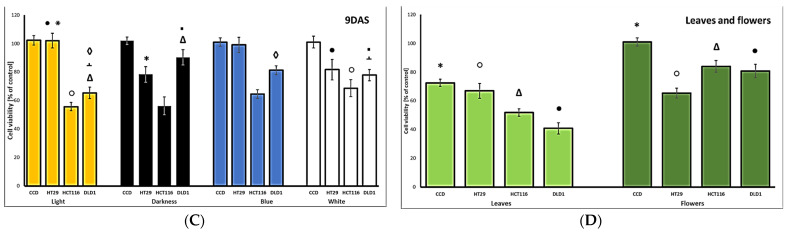
The cytotoxic effects of hemp sprout extracts (500 µg/mL) harvested after 5 (**A**), 7 (**B**), and 9 (**C**) days after sowing (DAS), under various lighting conditions (light, darkness, blue LED, white LED), and leaves or flowers (100 µg/mL) (**D**), on normal (CCD) and cancerous (HT29, HCT116, DLD1) colon cancer cells. Significant differences (*p* < 0.05) between different cell lines for each DAS or leaves and flowers are indicated by identical symbols.

The hemp products studied, namely leaves, flowers, and sprouts, represent functional food candidates, which could be implemented in the future as elements of dietary support in the chemoprevention of different diseases. Therefore, we decided to focus our study on gastrointestinal cells, including three colon cancer cell lines, differing in their origin and metastatic properties. To determine the selectivity of the tested samples, non-cancerous colon epithelial (CCD 841 CoN) cells were also included. The results of the cytotoxic activity of hemp sprouts at the highest concentration tested are shown in Figure 2A–C for 5, 7, and 9 days of harvesting, respectively. All sprouts’ extracts showed rather weak cytotoxic effect, with the exception of that observed for HCT-116 cells, which was moderate. Interestingly, out of the cell lines used in the study, HCT116 cells have the highest malignancy, and therefore the observed cytotoxic effect may be relevant in terms of chemoprevention. Less malignant DLD-1 cells were affected to a lesser extent, while HT29 cells, derived from a primary colon tumor, were the most resistant. However, with sprouts grown in darkness, the latter cell line was more susceptible than with sprout extracts grown under the other light conditions tested. The sprouts grown for 9 days revealed the most interesting and differential effect on the various colon cancer cells. Nevertheless, the results obtained for the 7DAS sprouts are also satisfactory. Importantly, none of the tested sprout samples was toxic to non-cancerous CCD 841 CoN colon epithelial cells, indicating their high selectivity and safety. It should be emphasized that this is the first reported cytotoxic effect of hemp sprouts, as there are no data on such an effect on any cell line.

Sofia-variety leaves showed a more pronounced cytotoxic effect compared to flowers (Figure 2D), with the exception of HT29 cells, on which the activity of both products was similar. Unlike sprouts, the leaves were most cytotoxic to DLD-1 cells, with IC_50_ 74 µg/mL, while the more malignant HCT116 cells were more resistant (IC_50_ 106 µg/mL). It should be noted that the advantage of the leaves over the flowers, observed at the concentrations above 50 µg/mL, was twofold for DLD-1 cells. However, it is still significantly weaker activity when compared to doxorubicin, used as a reference drug (IC_50_ 1.56–4.03 µg/mL). Importantly, both products were also safe for non-cancerous epithelial cells, while high toxicity of doxorubicin was noted (IC_50_ 1.67 µg/mL). A stronger cytotoxic effect of hexane extract from flowers of Eletta Campana hemp cultivar on HT29 and HCT116 cells was described [14], with an approximately 60% cell viability observed at 10 µg/mL, but this difference is most probably due to the solvent used for extraction, translating to the differences in the bioactive compounds found in the extract. Similarly, ethanolic flower extracts from six different Czech medical cannabis cultivars were highly cytotoxic to HT29 cells, but the authors related this activity to the presence of cannabinoids in the extracts [15]. On the other hand, other authors demonstrated high cytotoxicity of the polyphenolic fraction prepared from different Italian hemp varieties on the colon cancer cell lines Caco2 and SW480, thus pointing to cannflavins as the compounds responsible for the activity [16].

### 2.4. Anti-Inflammatory Activity

**Figure 3:**
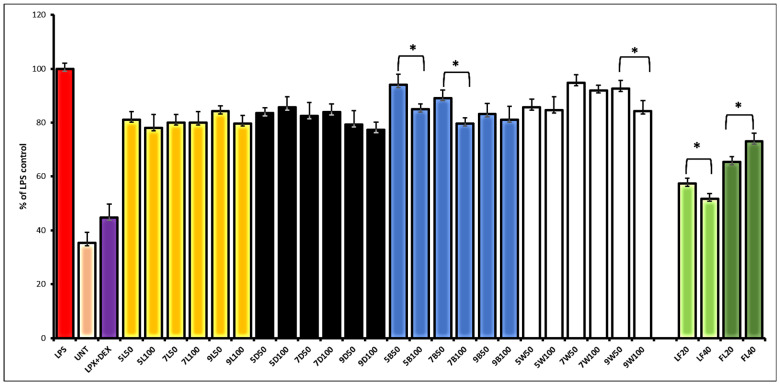
The effect of hemp sprouts harvested at different light conditions—light (L), darkness (D), blue LED (B), and white LED (W)—and leaves (LF) or flower (FL) extracts (50 and 100 µg/mL for sprouts; 20 and 40 µg/mL for LF and FL) on nitric oxide release in LPS-stimulated RAW 264.7 macrophages. Significant differences between the doses were marked by upper black line and * (*p* < 0.05).

As cancer development is often induced or enhanced by inflammation, in the final step of assessing chemopreventive properties, we examined whether the hemp products tested have anti-inflammatory potential. This was achieved by using an LPS-stimulated RAW 267.5 macrophage model, and the analysis was directed to the production of nitric oxide, the overproduction of which stimulates inflammation. Prior to the analysis, the effect of the extracts on macrophage viability was tested, and only two subcytotoxic doses of extracts were chosen for the anti-inflammatory assay, namely 50 and 100 µg/mL for sprouts, and 20 and 40 µg/mL for leaves and flowers. The results are shown in Figure 3. Among the sprout extracts, the best effect was observed for sprouts grown in the dark, followed by those grown under normal light conditions, where NO release decreased by about 20% compared to the control (LPS-stimulated cells). Although there were no significant differences between the doses used or the day of harvest, sprouts grown for 9 days in the dark appeared to be the most effective. Dose-dependent differences (*p* < 0.05) were noted for sprouts grown under blue (5B and 7B) or white (9W) LED light. Our results are the first to report the anti-inflammatory properties of hemp sprouts.

Similar to the observed cytotoxic effect, Sofia leaves’ extract also showed superior anti-inflammatory activity compared to the flowers, in a dose-dependent manner. Sofia leaves’ extract at the higher doses tested reduced NO release by up to approximately 50% relative to LPS-stimulated control cells, while a weaker effect of the flowers was observed at the higher dose tested compared to lower dose. We speculate that the high anti-inflammatory effect of the leaves might be related to the higher content of cannflavins, compared to the flowers, as some studies have described significant anti-inflammatory effects of these compounds [4]. On the other hand, Sofia flowers are rich in isoquercetin, which is also known for its anti-inflammatory properties. In a study by Zaiachuk et al. (2023), flower extracts of different cultivars, all rich in cannabinoids, strongly reduced the release of interleukins (IL-1β, 6, 8, 10) and TNF-alpha from LPS-stimulated RAW macrophages [17]. Similarly, cannabinoid-rich Thai hemp leaf extracts reduced not only NO release from LPS-stimulated fibroblasts, but also iNOS synthesis [18].

## 3. Materials and Methods

### 3.1. Reagents

Dimethyl sulfoxide (DMSO), chloroform, HPLC grade acetonitrile, water, and formic acid were purchased from Sigma-Aldrich (Seelze, Germany). Reference standards for HPLC analysis: chlorogenic acid, isochlorogenic acid, *p*-coumaric acid, ferulic acid, gallic acid, caffeic acid, quercetin, myricetin, isoquercetin, cannflavin A, and cannflavin B were purchased from LGC Standards. FeCl_3_·6H_2_O was from Sigma Chemical Co. (St. Louis, MO, USA), and 2,4,6-Trispyridyl-s-triazine (TPTZ) was purchased from Fluka Chemie (Buchs, Switzerland). Methanol, acetic acid, hydrochloric acid, sodium acetate, and sodium carbonate were from Avantor Performance Materials Poland S.A. (Gliwice, Poland). All reagents were of analytical grade. Distilled water was purchased from Sigma-Aldrich.

### 3.2. Plant Material and Sprout Harvesting

The seeds, dried leaves, and dried flowers of *Cannabis sativa* L. var. *sativa*, Sofia variety, were obtained from Polski Instytut Konopi (Lubań, Poland). Voucher specimens (KFg/2023/S1; KFg/2023/S2; KFg/2023/S3) were placed in the Department of Pharmacognosy Jagiellonian University Medical College. The seeds were sterilized with 70% ethanol for 5 min, and then soaked in water for 24 h. After that, the seeds were transferred to EQMM Easy Green Microfarm, and grown for 5, 7, and 9 days after seeding, at 25 °C, 70% humidity, in natural conditions of light (L), in total darkness (D), but also blue (B) and white (W) LED lights, being watered 3 times a day. The optimal photosynthetic photon flux density (PPFD) for cultivating hemp sprouts ranged from 100 to 300 µmol/m²/s. Briefly, the seeds were cultivated under blue LED light with a wavelength range of 430–505 nm (B) and under white LED light produced by the combination of greenish-blue light at 483 nm and orange light at 595 nm. Zeed LED lamps (Świętochłowice, Poland) were used. For the purpose of this manuscript, the obtained samples are denoted with appropriate numbers indicating the cultivation period followed by the acronym of the light conditions (e.g., 5D for sprouts harvested for 5 days in darkness).

### 3.3. Extraction and Quantitative Analysis

The fresh sprouts were Soxhlet-extracted with methanol, as previously described [19]. The samples of leaves and flowers were accurately weighed (2 g) and extracted with methanol in a water bath at 90 °C under reflux for 2 × 2 h. Extracts obtained were decanted, centrifuged, and stored in darkness in a freezer prior to HPLC analysis and antioxidative assays. The methanol extracts were further evaporated, and the dry residues were dissolved in DMSO and used for the cellular studies.

The quantitative analysis of flavonoids and phenolic acids in sprouts was performed, as previously described [7], on a Dionex HPLC system equipped with a PDA 100 UV–Vis detector and a Hypersil Gold (C-18) column (5 µm, 250 × 4.6 mm, Thermo EC). Analysis was carried out in a gradient mode, with 1% formic acid in water (A) and acetonitrile (B), 5–60% B in 60 min, at a flow rate of 1 mL/min. The detection wavelengths used were 254 and 285 nm. The compounds mentioned above were identified by comparing their retention times with those of the reference standards. The content of the individual compounds was calculated by measuring the peak area with respect to the appropriate standard curve (concentration range 0.0625–1 mg/mL). All analyses were performed in three replicates, and the mean value was expressed in mg/100 g of DW.

### 3.4. Determination of the Antioxidant Capacity

FRAP assay was performed according to modified method with 48-well plates and automatic reader (Synergy-2, BioTek Instruments Inc., Winooski, VT, USA), with syringe rapid dispensers as described previously [20]. To each plate, 0.4 mL of acetate buffer (pH 3.6) was dispensed, followed by 30 μL of different hemp extracts. The plate was conditioned at a temperature of 37 °C for 2 min, and then 0.2 mL of reagent mixture [1:1 (*v*/*v*) 20 mM ferric(III) chloride (FeCl_3_·6H_2_O) and 10 mM tripyridyltriazine in 40 mM HCl] was shaken for 30 s; afterwards, absorbance at 593 nm was measured with kinetic mode for 60 min. The method was calibrated using ferrous sulphate pentahydrate (FeSO_4_·5H_2_O) standard solution with a concentration range of 0–1.65 mM/L (0.27; 0.54; 0.82; 1.1; 1.37; 1.64 mM/L). The linearity range was assessed by plotting the analytical signal of ferrum against concentration and calculating the coefficient of determination (R^2^) by the method of least squares. An R^2^ of 0.999 indicated that the method was characterized by the linearity of the readings over the range of standard concentrations. The reducing ability of the sample was expressed in ferrous ion equivalents (micromolars of Fe^2+^ per g of plant dry weight) within the standard incubation period of 4.0 min after reagent addition. All analyses were performed in three replicates.

### 3.5. Cell Cultures and Viability Assay

Cytotoxic activity was tested on human colon adenocarcinoma HT29 (ATCC HTB-38), colon carcinoma HCT116 (ATCC CCL-247), colon adenocarcinoma DLD-1 (ATCC CCL 221), and normal human colon epithelial CCD 841 CoN (CRL 1790) cells. Cells were grown under standard conditions (37 °C, 5% CO_2_, relative humidity) and culture media (McCoy’s for HT29, DMEM high glucose for HCT116 and DLD-1), supplemented with 10% fetal bovine serum and 1% antibiotics solution (10,000 U penicillin and 10 mg streptomycin/mL). The stock solutions of the extracts were prepared in DMSO, and then diluted in the culture medium to the working concentrations (for sprouts 0–500 µg/mL, for leaves and flowers 0–100 µg/mL). Cell viability was determined after 24 h of incubation by MTT assay, as previously described [21]. Doxorubicin was used as a reference drug control. The absorbance was measured at 570 nm using a Biotek Synergy microplate reader (BioTek Instruments Inc., Winooski, VT, USA). All analyses were performed in three replicates, and the results are expressed as % of cell viability (mean ± SD) and IC_50_ values (concentration at which viability is inhibited by 50%) where possible.

### 3.6. Determination of Anti-Inflammatory Potential in RAW 264.7 Model

Murine RAW 264.7 macrophages were grown in DMEM high glucose, supplemented with 10% fetal bovine serum and 1% antibiotics solution. Before the experiment, the impact of the tested samples on RAW 264.7 cell viability was assessed using the MTT assay. For the anti-inflammatory assays, the cells were seeded onto 96-well plates (1.5 × 10^5^ cells/well) and pre-treated with the tested samples (50, 100 μg/mL for sprouts; 20, 40 μg/mL for leaves and flowers) or dexamethasone (0.5 μg/mL), a reference drug, for 1 h. Subsequently, 10 ng/mL of LPS was added to induce inflammation, as previously described [22]. The incubation was continued for the next 24 h. Cell culture supernatants were used for the determination of nitric oxide level, using Griess Reagent Kit (Promega Corporation, Madison, Winooski, VT, USA), according to the manufacturer’s protocol. The results were expressed as a percentage of the LPS control.

### 3.7. Statistical Analysis

The data were expressed as mean  ±  standard deviation (SD) and were analyzed using a one-way analysis of variance (ANOVA), along with a post hoc Tukey’s test; a Pearson correlation test was also used. Statistical analyses were conducted using STATISTICA v. 13.3. package (TIBCO Software Inc., Palo Alto, CA, USA).

## 4. Conclusions

This study highlights the influence of light conditions on phytochemical synthesis, contributing to the understanding of the health benefits of Sofia hemp products and their potential use in functional foods and pharmaceuticals. The results indicate the interesting chemopreventive potential of sprouts, leaves, and flowers from Sofia hemp variety, manifested as cytotoxic, antioxidant, and anti-inflammatory activity. Although the observed effects are interesting and promising, further studies are needed.

## Figures and Tables

**Table 1:** Content of phenolic acids and flavonoids, and antioxidant potential of sprouts of Sofia hemp variety, grown in different light conditions and harvested on different days after sowing (DAS) (n = 3).

Type of Light	DAS	Chlorogenic Acid [mg/100 g DW]	Caffeic Acid [mg/100 g DW]	Gallic Acid [mg/100 g DW]	Myricetin [mg/100 g DW]	FRAP μM Fe^2+^/g DW
Light	5	0.34 ± 0.01 ^ab^	0.24 ± 0.02 ^ab^	0.07 ± 0.01 ^ab^	0.09 ± 0.01 ^ab^	13.3 ± 2.3
	7	0.74 ± 0.05 ^cde^	0.09 ± 0.01 ^cd^	0.17 ± 0.01 ^cd^	0.18 ± 0.01 ^cd^	12.6 ± 2.0 ^a^
	9	0.09 ± 0.04 ^fg^	0.20 ± 0.01 ^e^	ND	0.08 ± 0.01 ^ef^	15.4 ± 1.9
Darkness	5	0.32 ± 0.01	0.16 ± 0.01 ^a^	0.09 ± 0.01	0.05 ± 0.02 ^a^	9.9 ± 1.2
	7	1.39 ± 0.03 ^c^	0.10 ± 0.04	0.26 ± 0.01 ^c^	0.28 ± 0.01^c^	9.5 ± 2.0
	9	0.42 ± 0.06 ^f^	0.23 ± 0.01	0.08 ± 0.01	0.19 ± 0.01 ^e^	14.0 ± 2.2
Blue	5	0.44 ± 0.01 ^a^	0.14 ± 0.01 ^b^	0.14 ± 0.01 ^a^	0.10 ± 0.01	11.7 ± 2.1
	7	0.48 ± 0.05 ^d^	0.31 ± 0.01 ^c^	0.13 ± 0.03	0.22 ± 0.01 ^d^	18.6 ± 1.9 ^a^
	9	0.11 ± 0.01	0.30 ± 0.01 ^e^	ND	0.07 ± 0.03	19.5 ± 2.4
White	5	0.66 ± 0.04 ^b^	0.23 ± 0.01	0.15 ± 0.02 ^b^	0.05 ± 0.01 ^b^	12.7 ± 1.8
	7	0.40 ± 0.02 ^e^	0.19 ± 0.03 ^d^	0.09 ± 0.02 ^d^	0.17 ± 0.01	17.0 ± 2.9
	9	0.21 ± 0.04 ^g^	0.23 ± 0.03	0.07 ± 0.01	0.11 ± 0.01 ^f^	19.8 ± 2.5

Myricetin measurements represent the amount of its derivatives in the samples. ND—not detected, DW—dry weight. Statistically significant differences (*p* < 0.05) were marked, for sprouts of the same DAS (days after sowing), in the columns with the same letters.

**Table 2:** Content of phenolic acids and flavonoids (mg/100 g DW), and antioxidant potential (μM Fe^2+^/g DW) in the methanolic extracts of leaves and flowers of the Sofia hemp variety.

Parameter	Leaves	Flowers
chlorogenic acid	3.61 ± 1.15	4.20 ± 1.26
isochlorogenic acid	14.97 ± 2.78 *	22.44 ± 1.82 *
*p*-coumaric acid	1.53 ± 0.19 *	2.37 ± 0.25 *
ferulic acid	2.41 ± 0.25 *	19.57 ± 4.19 *
gallic acid	ND	ND
cannflavin A	136.91 ± 5.99 *	76.35 ± 0.44 *
cannflavin B	3.28 ± 0.65	3.57 ± 0.14
quercetin	0.46 ± 0.072	ND
isoquercetin	21.78 ± 1.17 *	277.87 ± 39.31 *
myricetin	3.86 ± 0.3 *	25.85 ± 2.32 *
FRAP	185.16 ± 4.67 *	80.48 ± 3.14 *

Quercetin and myricetin measurements represent the amount of their derivatives in the samples. Statistically significant differences (*p* < 0.05) between leaves and flowers within each row were indicated by *.

## Data Availability

Data are contained within the article and Supplementary Materials.

## Supplementary Materials

The following supporting information can be downloaded at: https://www.mdpi.com/article/10.3390/plants13152023/s1, Figure S1. HPLC chromatogram of hemp sprouts extract; Figure S2. HPLC chromatogram of hemp leaves extract; Figure S3. HPLC chromatogram of hemp flowers extract.
