# Supplementary material for: Comparative Analysis of Polyphenolic Profile and Chemopreventive Potential of Hemp Sprouts, Leaves, and Flowers of the Sofia Variety
Source: Plants (Basel). 2024 Jul 23;13(15):2023. doi: 10.3390/plants13152023 (PMC11314196; doi:10.3390/plants13152023)
Supplement: Supplementary file 1 [file plants-13-02023-s001.zip › plants-3075478-supplementary.pdf]

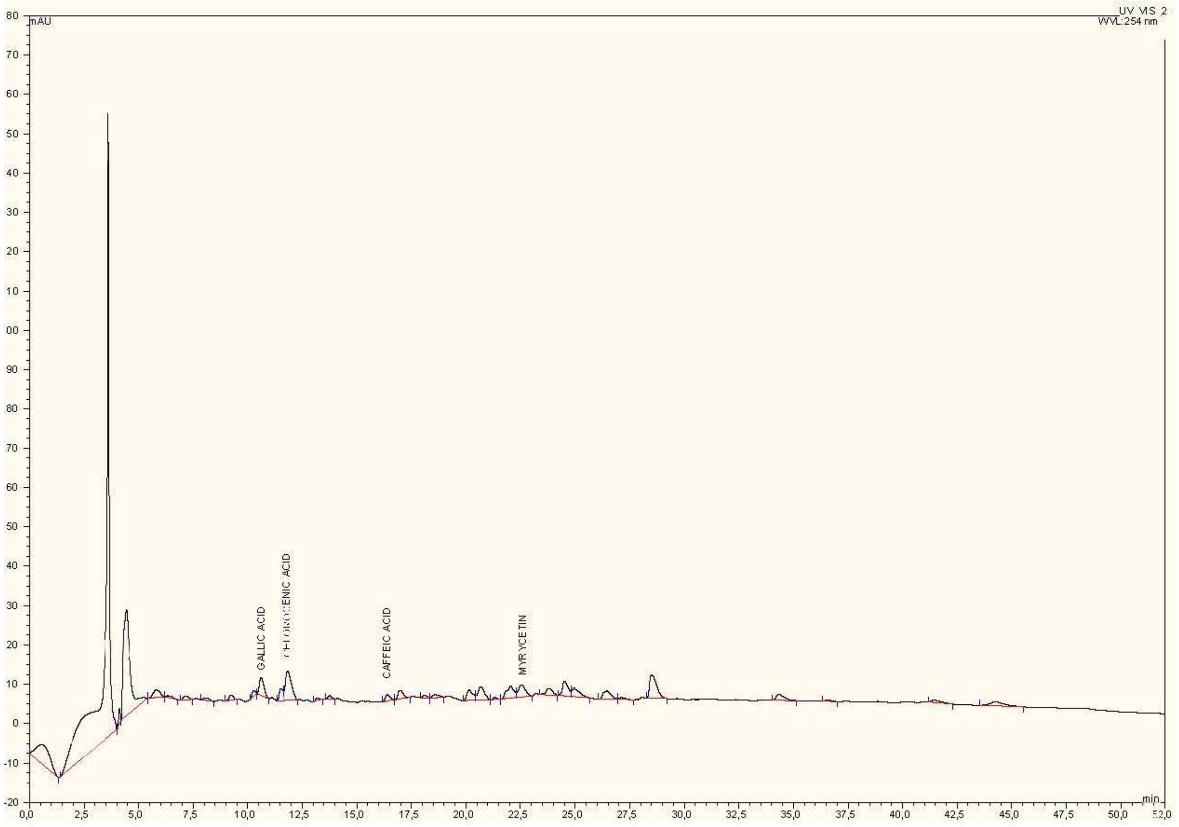

Figure S1. HPLC chromatogram of hemp sprouts extract.

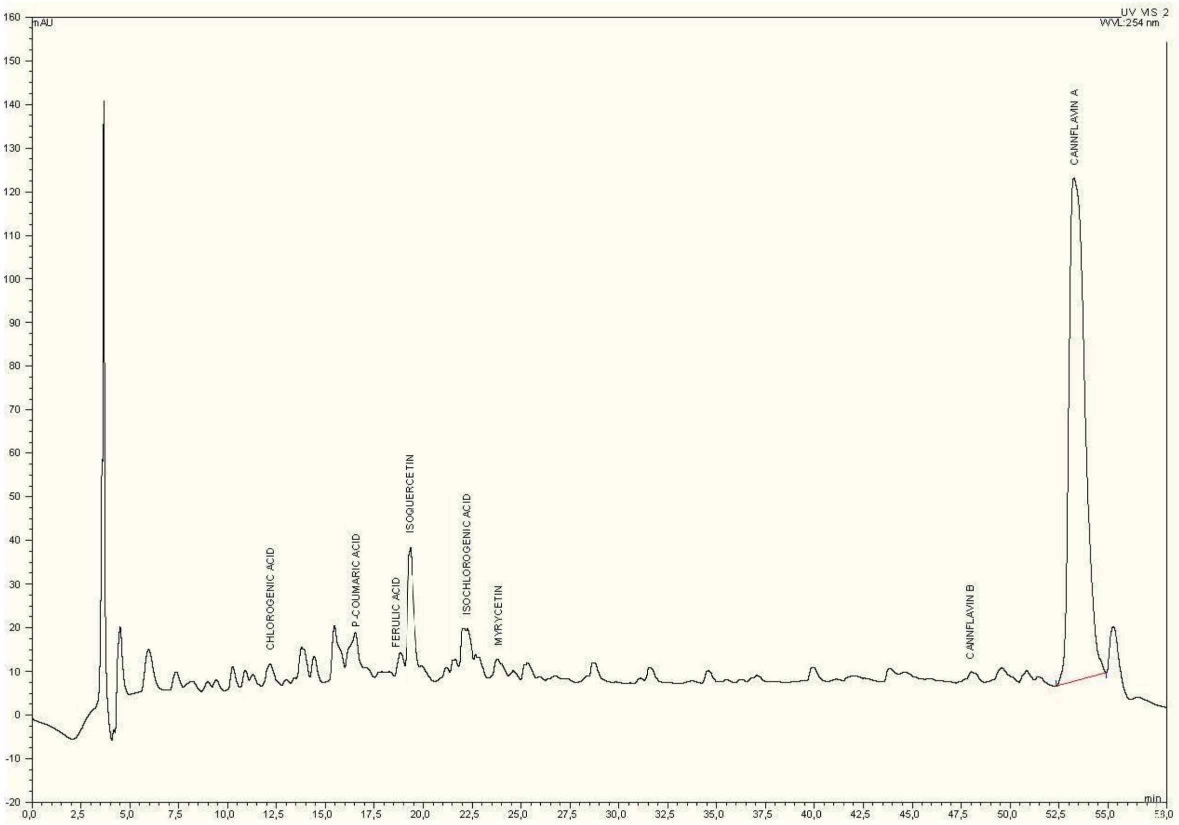

Figure S2. HPLC chromatogram of hemp leaves extract.

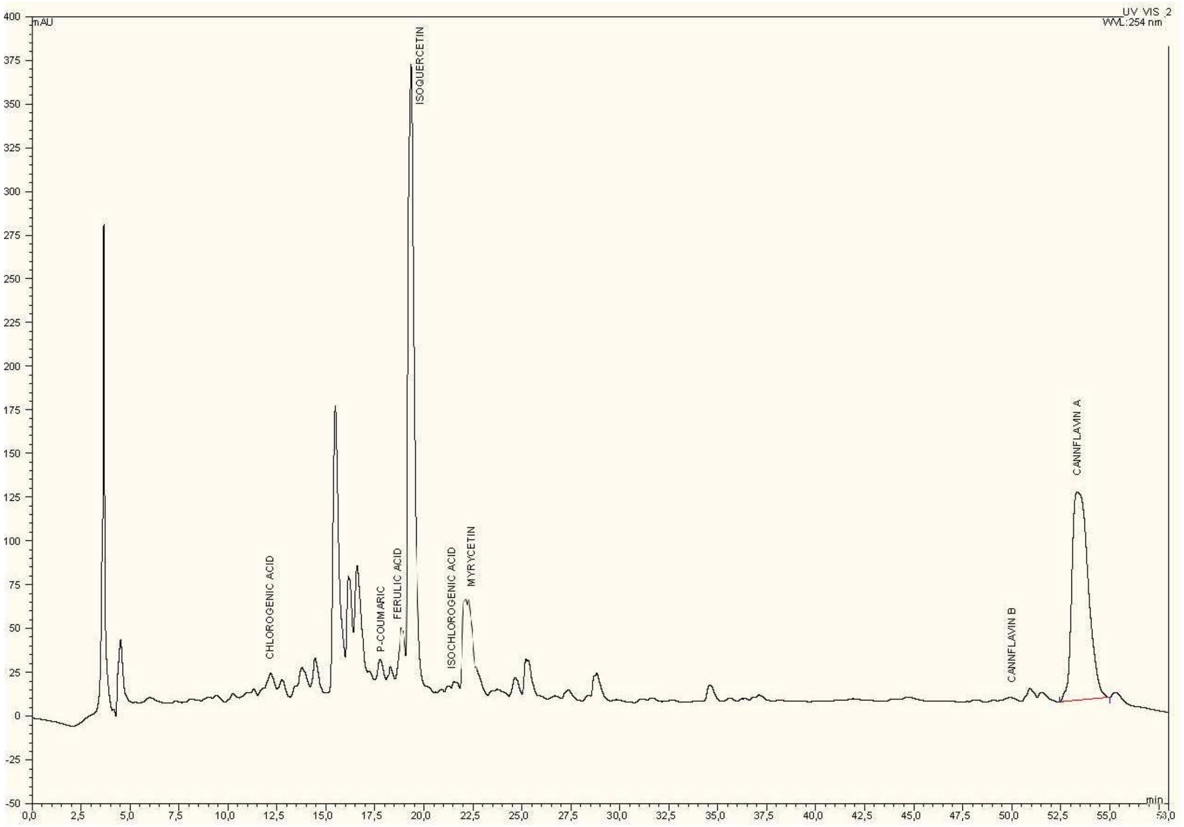

Figure S3. HPLC chromatogram of hemp flowers extract.
